# Growth in fossil and extant deer and implications for body size and life history evolution

**DOI:** 10.1186/s12862-015-0295-3

**Published:** 2015-02-14

**Authors:** Christian Kolb, Torsten M Scheyer, Adrian M Lister, Concepcion Azorit, John de Vos, Margaretha AJ Schlingemann, Gertrud E Rössner, Nigel T Monaghan, Marcelo R Sánchez-Villagra

**Affiliations:** Paläontologisches Institut und Museum der Universität Zürich, Karl Schmid-Strasse 4, CH-8006 Zürich, Switzerland; Department of Earth Sciences, The Natural History Museum, Cromwell Road, London, SW7 5BD UK; Department of Animal and Vegetal Biology and Ecology, Faculty of Experimental Sciences, University of Jaén, Jaén, 23071 Spain; Naturalis Biodiversity Center, Postbus 9517, 2300 RA Leiden, The Netherlands; Department of Integrative Zoology, IBL, Leiden University, Sylviusweg 72, Postbus 9505, 2300 RA Leiden, The Netherlands; Bayerische Staatssammlung für Paläontologie und Geologie, Richard-Wagner-Strasse 10, D-80333 München, Germany; National Museum of Ireland-Natural History, Merrion Street, Dublin 2, Ireland

**Keywords:** Island evolution, Pleistocene, Cervidae, *Candiacervus*, *Megaloceros*, Bone histology, Cementum analysis, Growth rates, Longevity, Skeletal maturity

## Abstract

**Background:**

Body size variation within clades of mammals is widespread, but the developmental and life-history mechanisms by which this variation is achieved are poorly understood, especially in extinct forms. An illustrative case study is that of the dwarfed morphotypes of *Candiacervus* from the Pleistocene of Crete versus the giant deer *Megaloceros giganteus*, both in a clade together with *Dama dama* among extant species*.* Histological analyses of long bones and teeth in a phylogenetic context have been shown to provide reliable estimates of growth and life history patterns in extant and extinct mammals.

**Results:**

Similarity of bone tissue types across the eight species examined indicates a comparable mode of growth in deer, with long bones mainly possessing primary plexiform fibrolamellar bone. Low absolute growth rates characterize dwarf *Candiacervus* sp. II *and C. ropalophorus* compared to *Megaloceros giganteus* displaying high rates, whereas *Dama dama* is characterized by intermediate to low growth rates. The lowest recorded rates are those of the Miocene small stem cervid *Procervulus praelucidus*. Skeletal maturity estimates indicate late attainment in sampled *Candiacervus* and *Procervulus praelucidus*. Tooth cementum analysis of first molars of two senile *Megaloceros giganteus* specimens revealed ages of 16 and 19 years whereas two old dwarf *Candiacervus* specimens gave ages of 12 and 18 years.

**Conclusions:**

There is a rich histological record of growth across deer species recorded in long bones and teeth, which can be used to understand ontogenetic patterns within species and phylogenetic ones across species. Growth rates *sensu* Sander & Tückmantel plotted against the anteroposterior bone diameter as a proxy for body mass indicate three groups: one with high growth rates including *Megaloceros*, *Cervus*, *Alces*, and *Dama*; an intermediate group with *Capreolus* and *Muntiacus*; and a group showing low growth rates, including dwarf *Candiacervus* and *Procervulus*. Dwarf *Candiacervus*, in an allometric context, show an extended lifespan compared to other deer of similar body size such as *Mazama* which has a maximum longevity of 12 years in the wild. Comparison with other clades of mammals reveals that changes in size and life history in evolution have occurred in parallel, with various modes of skeletal tissue modification.

**Electronic supplementary material:**

The online version of this article (doi:10.1186/s12862-015-0295-3) contains supplementary material, which is available to authorized users.

## Background

Several lineages of mammals have evolved remarkable changes in body size following island isolation [1-3], including among others dwarf hippopotamuses, elephants, and deer, and giant rabbits [4-6]. These patterns are the result of complex interplay of multiple variables, including resource limitation and ecological release [5,7-9]. To understand the mechanisms of life-history and size evolution on islands but also in cases of significant body size changes in mainland lineages, histology of hard tissues is a powerful tool, as has been demonstrated for ‘dwarf’ and ‘giant’ sauropod [10-12] and tyrannosaurid [13] dinosaurs, as well as early synapsids [14,15] among fossil forms.

A remarkable example of island evolution is found in the Pleistocene of Crete, where an endemic clade of deer, *Candiacervus*, including ‘dwarfed’ species, evolved from the megacerine clade (Megacerini) of larger forms [16-19]. Despite of the unresolved nature of megacerine phylogeny [20], the small *Candiacervus* morphotypes must have undergone size reduction since all their postulated mainland sister-groups are significantly larger (e.g. *Praemegaceros spp.* with shoulder heights ranging from 0.9 m to 1.50 m [18,19] or *Cervus peloponnesiacus* with a shoulder height of just slightly less than one metre [21]. The kind of dwarfism we observe in *Candiacervus* has been described as autapomorphic nanism by [22]. *Candiacervus* shows diversity in size, as six size classes of deer have been distinguished [16,17]. The smallest morphotype, *C. ropalophorus*, reached a shoulder height of about 40 cm, *C*. sp. II one of about 60 cm, and the largest one reached a height of about 1.65 m [23]. This phenomenon has been interpreted as a case of adaptive radiation [24]. In the Middle to Late Pleistocene, Crete was characterized by dense forest as well as jagged rocks with several intermediate kinds of environments, in which such a radiation could have occurred [5]. Here we study *Candiacervus ropalophorus* and *C*. sp. II, as these two size classes are small and are represented by growth series we could sample. ‘*Candiacervus* sp. II’ may be a composite of three morphotypes of similar size [17].

**Figure 1:**
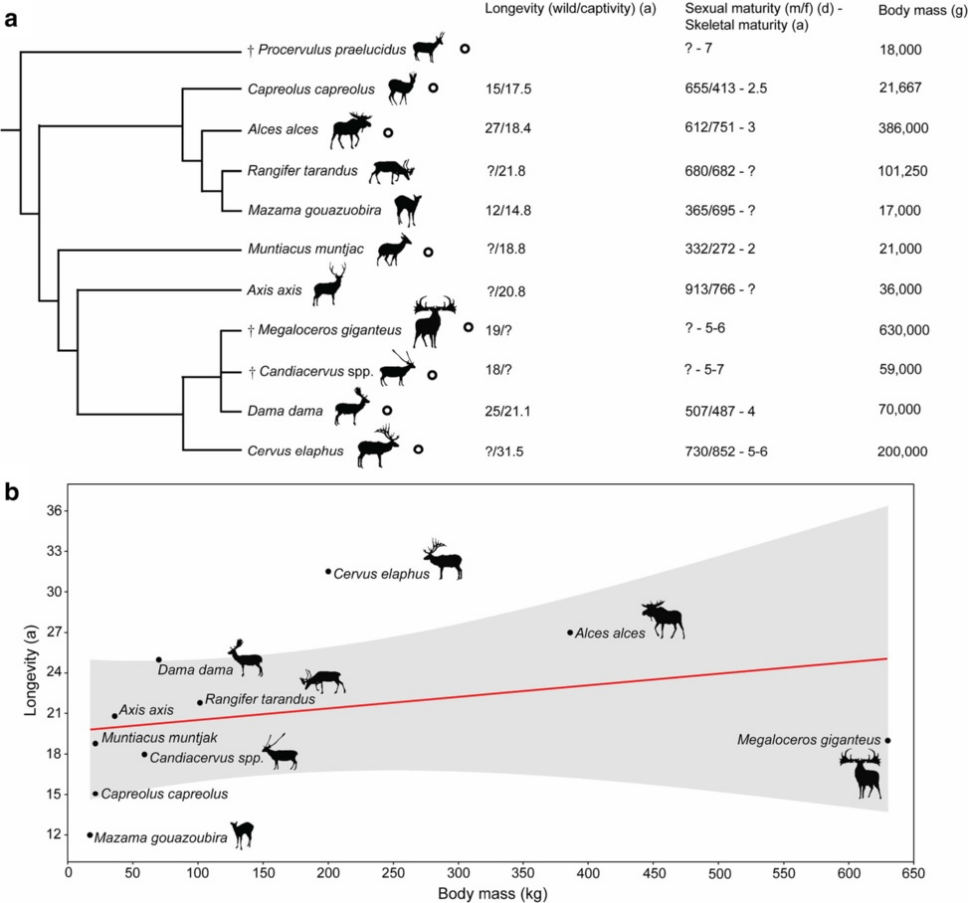
**Cervid phylogeny and life history. a)** Phylogenetic relationships, maximal recorded life expectancy, onset of maturity, and average adult body mass of *Candiacervus*, *Megaloceros*, and relatives based on [17,20,23,28-35]. Cervid species studied are indicated by black circles. The trichotomy of the fallow deer (*Dama*) and its extinct relatives illustrates the unresolved nature of this well-supported clade. Body mass values are average. **b)** Linear regression relating maximum recorded longevity to body mass in cervids, and observed maxima within available samples of fossil species. Shaded region represents the 95% confidence interval. In cases in which unambiguous longevity data for wild animals were not available, data of captive specimens have been used.

Representing the other extreme of size with a shoulder height of up to 2 m [20], *Megaloceros giganteus* has been a subject of extensive debates on evolutionary processes [20,25,26]. It is best known from fossil occurrences in Ireland from 11 to 12,000 BP [27] years ago and from possessing the largest antlers of any fossil or living species. *Megaloceros* was widespread in Europe and western Asia for 400,000 years and morphological and molecular analyses have supported a close relationship with fallow deer, *Dama dama* [20,28] (Figure 1a). The fossil record of deer is long and complex, and *Procervulus praelucidus* from the Early Miocene of Germany represents a stem taxon that can help to reconstruct the evolution of life history features in deer [29] (Figure 1a).

In order to enhance reproductive success life history traits can be selected by adjusting the developmental schedule to match environmental conditions [36]. Bone microstructure can reveal such traits in mammals, which generally exhibit bone matrices indicative of high rates of tissue deposition in juveniles, whereas after onset of maturity a decrease in bone growth rate occurs resulting in deposition of highly organized bone tissue [37-39]. Lines of arrested growth (LAGs) form from the first year of an individual’s postnatal life as a result of annual cessation of bone growth [40,41]. Counting these LAGs therefore provides the means to estimate minimum individual ages [40]. However, there can be decoupling of the number of LAGs in long bones and the actual age of old individuals leading to underestimation of individual ages [42]. Dental cementum is a more accurate source for estimating longevity in mammals, due to its usual absence of resorption [43] and more complete growth record, as shown by studies on growth marks in living species, including deer of known age [43,44]. For example, using cementum analysis in molars, 99% of a sample of 51 Spanish red deer could be aged within a one year confidence interval [45].

Palaeohistology previously led to the discovery in the island goat *Myotragus balearicus* from the Late Pleistocene of the Balearic Islands, of a ‘reptile’-like growth pattern consisting of lamellar-zonal bone throughout the cortex [46]. *Myotragus* was therefore hypothesized to have grown at low but variable rates and to have ceased its growth cyclically. Our investigation of *Candiacervus* and of relevant mainland cervids, focusing on bone microstructure in growth series of various long bones, and dental histology in old adults, serves to examine whether the pattern of growth of *Myotragus* is general among island artiodactyls. Longevity estimates, based on the rest lines in the first molar of old individuals, were made. The first molar is the first permanent tooth to erupt [45], showing the most complete growth record in deer. In order to further examine growth patterns across cervids and to put life history data attained by histological analyses into an allometric context, we investigated the relation between body weight and growth rates [47-49].

## Methods

A total of 51 long bones, six phalanges, four lower first molars and two upper first molars of *Candiacervus* sp. II and *Candiacervus ropalophorus*, 14 long bones and five lower first molars of *Megaloceros giganteus*, and 13 long bones and 2 lower first molars of *Procervulus praelucidus* were sampled (Table 1, see also Additional file 1: Methods). Sixteen long bones and two lower first molars of *Dama dama*, and one femur each of *Muntiacus muntjak*, *Cervus elaphus* and *Alces alces*, were sampled for comparison. Of *Capreolus capreolus* one femur and one metacarpal were sampled. Following standard procedures, the bones were coated and impregnated with epoxy resin (Araldite or Technovit) prior to sawing and grinding. Long bones were transversely sectioned at mid-shaft where the growth record is most complete [e.g. 10]. For cementum analysis jaws were longitudinally cut through the cementum interroot pad of the lower first molar and surfaces were impregnated with epoxy resin and finally ground and polished. Long bones of *Megaloceros giganteus* were also sampled by using a diamond-studded core drill, with sampled cores being subsequently processed [10,50]. Sections were observed in normal transmitted and cross-polarized light using a Leica DM 2500 M composite microscope equipped with Leica DFC 420 C digital camera. Since there are no remarkable differences in the bone tissue of the two *Candiacervus* morphotypes sampled, they are treated together here. Polished tooth surfaces were observed using a Leica MZ 165 and MZ 125 reflected-light microscope.

**Table 1:** **Material used in this study**

**Species**	**Object**	**Ontogenetic stage**	**Locality**	**Specimen number**
*Candiacervus ropalophorus*	Femur	adult	Gerani 4, Crete (Greece)	PIMUZ A/V 5195
"	"	adult	"	PIMUZ A/V 5202
"	"	perinatal	"	PIMUZ A/V 5207
"	"	perinatal	"	PIMUZ A/V 5206
"	Tibia	adult	"	PIMUZ A/V 5188
"	"	adult	"	PIMUZ A/V 5189
"	"	juvenile	"	PIMUZ A/V 5208
"	"	juvenile	"	PIMUZ A/V 5193
"	"	perinatal	"	PIMUZ A/V 5191
"	"	perinatal	"	PIMUZ A/V 5194
"	Metatarsus	adult	"	PIMUZ A/V 5192
"	"	juvenile	"	PIMUZ A/V 5254
"	"	perinatal	"	PIMUZ A/V 5205
"	Humerus	adult	"	PIMUZ A/V 5190
"	"	perinatal	"	PIMUZ A/V 5187
"	"	perinatal	"	PIMUZ A/V 5203
"	Radius	adult	"	PIMUZ A/V 5186
"	"	adult	"	PIMUZ A/V 5199
"	"	perinatal	"	PIMUZ A/V 5200
"	Ulna	perinatal	"	PIMUZ A/V 5255
"	Metacarpus	adult	"	PIMUZ A/V 5197
"	"	juvenile	"	PIMUZ A/V 5198
"	Lower M1	adult	"	PIMUZ A/V 5196
*Candiacervus* sp. II	Femur	adult	Liko, Crete (Greece)	PIMUZ A/V 5218
"	"	juvenile	"	PIMUZ A/V 5219
"	"	perinatal	"	PIMUZ A/V 5244
"	"	perinatal	"	PIMUZ A/V 5245
"	Tibia	adult	"	PIMUZ A/V 5222
"	"	juvenile	"	PIMUZ A/V 5220
"	"	perinatal	"	PIMUZ A/V 5221
"	"	perinatal	"	PIMUZ A/V 5234
"	Metatarsus	adult	"	PIMUZ A/V 5240
"	"	adult	"	PIMUZ A/V 5212
"	"	juvenile	"	PIMUZ A/V 5213
"	"	juvenile	"	PIMUZ A/V 5223
"	"	perinatal	"	PIMUZ A/V 5224
"	Humerus	adult	"	PIMUZ A/V 5231
"	"	juvenile	"	PIMUZ A/V 5236
"	"	perinatal	"	PIMUZ A/V 5237
"	Radius	adult	"	PIMUZ A/V 5232
"	"	adult	"	PIMUZ A/V 5233
"	"	juvenile	"	PIMUZ A/V 5230
"	"	perinatal	"	PIMUZ A/V 5211
"	"	perinatal	"	PIMUZ A/V 5257
"	"	perinatal	"	PIMUZ A/V 5214
"	Ulna	adult	"	PIMUZ A/V 5215
"	"	juvenile	"	PIMUZ A/V 5225
"	"	perinatal	"	PIMUZ A/V 5226
"	Metacarpus	adult	"	PIMUZ A/V 5246
"	"	juvenile	"	PIMUZ A/V 5247
"	"	perinatal	"	PIMUZ A/V 5209
"	"	perinatal	"	PIMUZ A/V 5210
"	1st Phalange	adult	"	PIMUZ A/V 5238
"	"	juvenile	"	PIMUZ A/V 5239
"	"	perinatal	"	PIMUZ A/V 5216
"	2nd Phalange	adult	"	PIMUZ A/V 5217
"	"	juvenile	"	PIMUZ A/V 5235
"	"	perinatal	"	PIMUZ A/V 5227
"	Rib	adult	"	PIMUZ A/V 5228
"	Lower M1	adult	"	PIMUZ A/V 5229
"	"	adult	"	PIMUZ A/V 5243
"	Upper M1	senescent (18 years)	"	PIMUZ A/V 5241
"	"	adult	"	PIMUZ A/V 5242
*Candiacervus* sp.	Lower M1	senescent (12 years)	Bate cave, Crete (Greece)	PV M 82318 (NHML)
*Procervulus praelucidus*	Femur	adult	Wintershof-West, Germany	BSPG 1937 II 23226
"	"	adult	"	BSPG 1937 II 23227
"	"	juvenile	"	BSPG 1937 II 23228
"	"	juvenile	"	BSPG 1937 II 23229
"	Tibia	adult	"	BSPG 1937 II 23230
"	"	adult	"	BSPG 1937 II 23231
"	"	juvenile	"	BSPG 1937 II 23232
"	Humerus	adult	"	BSPG 1937 II 23233
"	"	adult	"	BSPG 1937 II 23234
"	Radius	adult	"	BSPG 1937 II 23235
"	"	adult	"	BSPG 1937 II 23236
"	"	adult	"	BSPG 1937 II 23237
"	"	adult	"	BSPG 1937 II 23238
"	Lower M1	adult	"	BSPG 1937 II 12002
"	"	adult	"	BSPG 1937 II 12040
*Megaloceros giganteus*	Femur	adult	Craddanstown Rep. of Ireland	NMING:F7937/4
"	"	adult	Baunmore Townland, Rep. of Ireland	NMING:F21306/13
"	Tibia	adult	Ballyragget, Rep. of Ireland	NMING:F22655/34
"	"	adult	Buttevant, Rep. of Ireland	NMING:F22534/5
"	"	adult	Baunmore Townland, Rep. of Ireland	NMING:F21306/14
"	Metatarsus	adult	North Sea sediments	PIMUZ A/V 5256
"	"	adult	Baunmore Townland, Rep. of Ireland	NMING:F21306/19
"	"	adult	Buttevant, Rep. of Ireland	NMING:F22534/6
"	Humerus	adult	Ballyragget, Rep. of Ireland	NMING:F22655/37
"	"	adult	Buttevant, Rep. of Ireland	NMING:F22534/2
"	Radius-Ulna	adult	Ballyragget, Rep. of Ireland	NMING:F22655/36
"	"	adult	Buttevant, Rep. of Ireland	NMING:F22534/3
"	Metacarpus	adult	Ballyragget, Rep. of Ireland	NMING:F22655/31
"	"	adult	Buttevant, Rep. of Ireland	NMING:F22534/4
"	Lower M1	senescent (19 years)	Brühl (Schwetzingen), Deutschland	PIMUZ A/V 2235
"	"	senescent (16 years)	Kent'scavern, Torquay, UK	PV OR 16800 (NHML)
"	"	senescent (n.a.)	Wyhlen, Germany	BSPG 1957 I 398
"	"	adult	Rath, Rep. of Ireland	NMING:F22654
"	"	adult	Craddanstown, Rep. of Ireland	NMING:F7937/5
*Dama dama*	Femur	adult (wild)	Schrevenborn, Germany	ZIUK 9630
"	"	adult (captive)	Wildnispark Zürich, Switzerland	PIMUZ A/V 5248
"	"	adult (captive)	"	PIMUZ A/V 5248
"	"	juvenile (captive)	"	PIMUZ A/V 5249
"	Tibia	adult (wild)	Schrevenborn, Germany	ZIUK 9630
"	"	adult(captive)	WildnisparkZürich, Switzerland	PIMUZ A/V 5248
"	"	adult (captive)	"	PIMUZ A/V 5248
"	"	juvenile (captive)	"	PIMUZ A/V 5249
"	Humerus	adult (wild)	Schrevenborn, Germany	ZIUK 9630
"	"	adult (captive)	Wildnispark Zürich, Switzerland	PIMUZ A/V 5248
"	"	adult (captive)	"	PIMUZ A/V 5248
"	"	juvenile (captive)	"	PIMUZ A/V 5249
"	Radius-Ulna	adult (wild)	Schrevenborn, Germany	ZIUK 9630
"	"	adult (captive)	Wildnispark Zürich, Switzerland	PIMUZ A/V 5248
"	"	adult (captive)	"	PIMUZ A/V 5248
"	"	juvenile (captive)	"	PIMUZ A/V 5249
"	Lower M1	adult (wild)	Schrevenborn, Germany	ZIUK 9630
"	"	adult (captive)	Wildnispark Zürich, Switzerland	PIMUZ A/V 5248
*Capreolus capreolus*	Femur	adult (wild)	Schrevenborn, Germany	ZIUK 9872
"	Metatarsus	juvenile (wild)	Hittnau, Switzerland	PIMUZ A/V 5251
*Muntiacus muntjak*	Femur	adult (captive)	Tierpark Hagenbeck, Hamburg, Germany	ZIUK 7994
*Cervus elaphus*	"	adult (wild)	Barmstedt, Germany	ZIUK 23517
*Alces alces*	"	adult (wild)	Norway	ZMUZ 20242

Specimens used in this study with ontogenetic stage, locality of death/fossil site, specimen number and thin section number.

Institutional Abbreviations: **BSPG** Bayerische Staatssammlung für Paläontologie und Geologie, Munich, Germany; **NBC** Netherlands Centre for Biodiversity Leiden, The Netherlands; **NHML** Natural History Museum London, UK; **NMING** National Museum of Ireland - Natural History; **PIMUZ** Paläontologisches Institut und Museum, Universität Zürich, Switzerland; **ZIUK** Zoologisches Institut der Universität Kiel, Germany; **ZMUZ** Zoologisches Museum der Universität Zürich, Switzerland.

For quantification of growth rates, distances between LAGs, i.e. growth zones were measured with Leica IM 50 Image Manager®, and annual growth rates per day were calculated [51] by dividing growth zones by the number of days per growth period and year. The estimate of number of days per growth period, i.e. 260 days, is based on [41]. Growth period intervals (275–245 days) [41] and a 365 day growth period have been taken into account as well (Additional file 2). Since growth zone thickness may vary considerably within the cortex of one bone, all measurements have been performed along the anteroposterior axis in the anterior quadrant of each section, whereas micrographs presented in this work have been taken from the best preserved and histologically most informative areas. Growth zone measurements were performed for femora and tibiae since they are the most informative long bones in cervids (see also Additional file 1: Methods). For growth rate graphs Microsoft Office Excel 2010^©^ has been used. Regression analyses (ordinary least squares) for average and growth rates *sensu* Sander & Tückmantel [51] were performed using Past3.0 [52]. All graphs have been redrawn using Adobe Illustrator CS5^©^_._

## Results and discussion

### Histological description of primary bone

**Figure 2:**
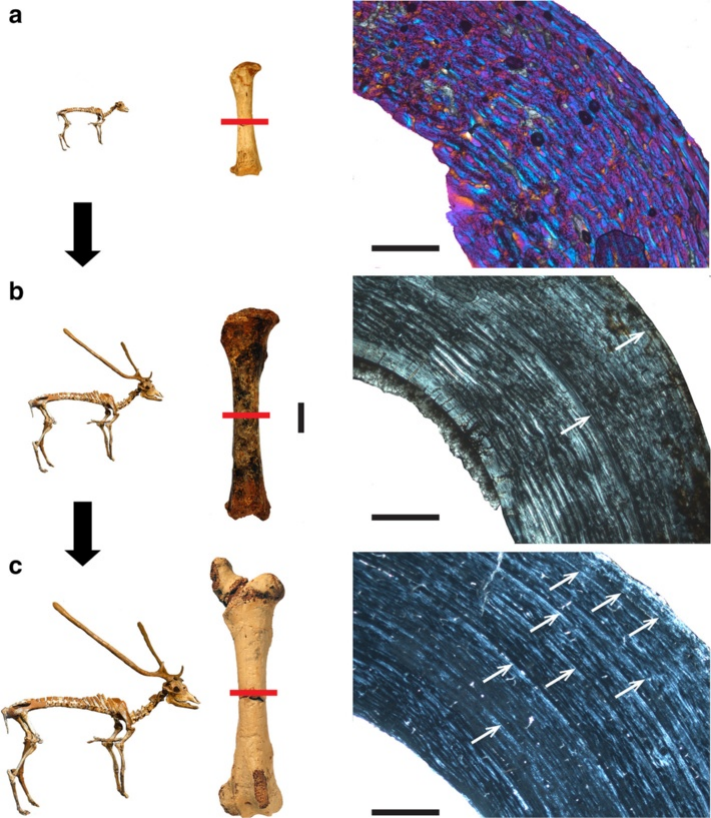
**Histological growth series of dwarf deer femora with skeletal reconstructions** **[** 55 **]** **and specimens sampled (anterior view).** Red bars indicate plane of sectioning. *Candiacervus* sp. II under crossed polarised light (xpl). **a)** Bone cortex of a perinatal specimen (PIMUZ A/V 5244) showing a mainly plexiform arrangement of vascular canals with a high amount of woven-fibred bone (violet areas; additional use of lambda compensator). Scale bar, 0.5 mm. **b)** Juvenile specimen (PIMUZ A/V 5219). Scale bars, 20 mm (femora all to scale), 1 mm (bone cortex). **c)** Adult specimen (PIMUZ A/V 5218). Note the increasing amount of parallel-fibred bone (bright areas) and the occurrence of lines of arrested growth (LAGs, white arrows) throughout ontogeny. Scale bar, 1 mm.

Newborn dwarf *Candiacervus* (*C. ropalophorus* and *C.* sp. II) exhibit fibrolamellar bone with a high amount of woven-fibred bone as primary tissue (Figure 2a). In the inner cortex, vascularization tends to be reticular, whereas in the middle and outer cortex vascularization has a plexiform pattern. With increasing age, the amount of vascularisation and woven bone decreases, with the former changing from a plexiform to laminar organisation in the middle and outer cortex, whereas the amount of lamellar or parallel-fibred bone within the fibrolamellar matrix increases (Figure 2a-c). The outermost layer of the outer cortex in adult *Candiacervus* sampled is composed of a narrow layer of avascular lamellar bone, called the outer circumferential layer (OCL) [53] in this work and also referred to as external fundamental system (EFS, e.g. *sensu* [42], see also [54]). We prefer the term outer circumferential layer for being more descriptive than the term external fundamental system. An inner circumferential layer [38] is well developed in all adult femora. Long bones of *Candiacervus* indicate, based on growth line counts, minimum ages of about two years for the juveniles sampled.

**Figure 3:**
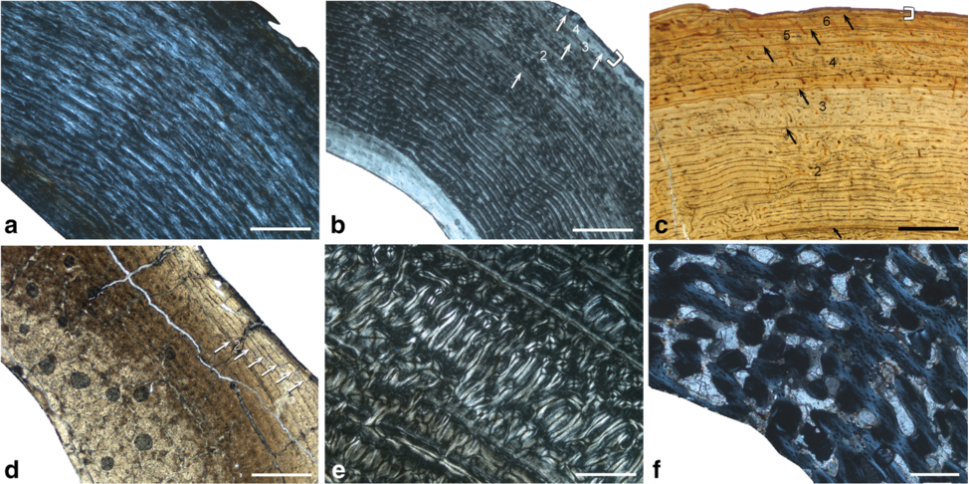
**Cervid bone tissue and growth marks.** Increasing femoral vascularisation **(a-c)** accompanied by increasing body size in a) juvenile *Procervulus* specimen BSPG 1937 II 23227, medial cortex (xpl; scale bar, 0.5 mm), **b)** adult *Dama* specimen ZIUK 9630, medial cortex (xpl; scale bar, 1 mm), and **c)** adult *Megaloceros* specimen NMING: F21306/13 under plane polarised light, anterior cortex (ppl, scale bar 1 mm). Note the low amount of vascularisation in the fibrolamellar bone of the juvenile *Procervulus*. Occurrence of LAGs indicated by black/white arrows and the outer circumferential layer by white brackets. Numbers indicate growth zones. Bone surfaces at the top, medullary cavities at bottom left. **d)** Tibia of adult *Candiacervus* sp. II (PIMUZ A/V 5222) showing Haversian bone in the inner part of the posterior cortex (bright area) and plexiform fibrolamellar bone in the middle part (lpl; scale bar, 1 mm). Occurrence of LAGs indicated by white arrows. Bone surface at top right, medullary cavity at bottom left. **e)** Radiating fibrolamellar bone in a metacarpal of adult *Megaloceros giganteus* (NMING:F22534/4, xpl; scale bar, 0.5 mm). **f)** First phalange of perinatal *Candiacervus* sp. II (PIMUZ A/V 5216) showing reticular vascularisation of mainly woven-fibred bone (xpl; scale bar, 0.2 mm).

Adult *Megaloceros*, *Dama*, *Cervus*, and *Alces* show in all sampled long bones a similar arrangement of bone tissue types to each other. Vascularisation in the outer part of the cortex is partly longitudinal, whereas in dwarf *Candiacervus*, *Procervulus*, and *Muntiacus* it changes directly from plexiform/laminar to avascular in the outer circumferential layer. This is evidently a feature separating large and intermediate from small-sized deer, including dwarf *Candiacervus*. Moreover, the density of vascular canals is higher in intermediate-sized and larger deer compared to smaller taxa (Figure 3a-c, see also Additional file 1: Table S2 and Figure S1). Because it is a juvenile specimen and still shows less vascularisation than the adult *Dama* and *Megaloceros*, specimen BSPG 1937 II 23227 is especially illustrative concerning the low amount of vascularization in the small sized cervid *Procervulus* (Figure 3a-c).

**Figure 4:**
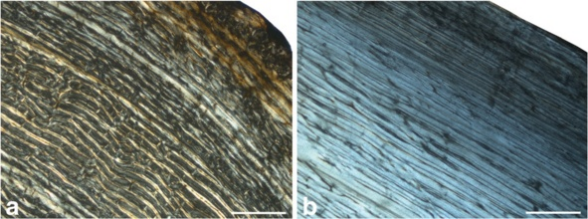
**Bone cortex of** ***Megaloceros giganteus*** **.** Femur (NMING: F21306/13) in transverse **(a)** and longitudinal **(b)** section under crossed polarised light (bone surface top right). Note the low amount of woven fibred bone (dark areas) in the longitudinal section.

A longitudinal section of a *Megaloceros* femur confirms the low amount of woven-fibred bone in plexiform bone tissue [56] (Figure 4a,b). However, since woven-fibred bone is present, we follow [57] in using the term fibrolamellar bone. In general, the bone tissue found in the femora and humeri gives a similar picture. The differences in the amount of vascularisation observed in dwarf *Candiacervus* and large/intermediate sized cervids are less obvious in the humeri than those seen in the femora. Unlike *Candiacervus* (Figure 3d), tibiae of *Dama* and *Megaloceros* show areas of radiating fibrolamellar bone interdigitating with the otherwise plexiform bone tissue. In general, adult radii of all sampled deer have a similar arrangement of bone tissue types, i.e. plexiform fibrolamellar bone with a varying amount of Haversian bone. The amount of woven bone of perinatal ulnae of dwarf *Candiacervus* is high in the inner cortex.

Similarity of bone tissue types in *Candiacervus*, *Megaloceros*, and *Capreolus* shows a comparable mode of growth in the metapodials. A remarkable difference distinguishing adult *Megaloceros* from *Candiacervus* is the occurrence of layers of radiating fibrolamellar bone (Figure 3e) in the middle and outer cortex of *Megaloceros*. The inner circumferential layer is relatively thicker in *Megaloceros* than in *Candiacervus*.

Phalanges of newborn *Candiacervus* specimens show fibrolamellar bone with reticular vascularisation (Figure 3f). During ontogeny, the vascular organisation becomes plexiform, but with increasing age this is replaced by increasing amounts of poorly vascularised lamellar/parallel-fibred bone in the sub-periosteal region.

### Secondary bone and remodelling processes

**Figure 5:**
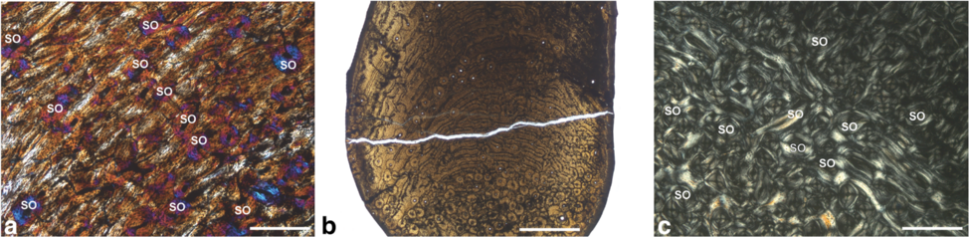
**Cervid bone remodelling. a)** Humerus of juvenile *Candiacervus* sp.II specimen PIMUZ A/V 5236 (xpl, lambda compensator, scale bar 0.5 mm). Note the scattered secondary osteons (SO). **b)** Ulna of adult *Candiacervus* sp. II specimen PIMUZ A/V 5215 (lpl, scale bar 1 mm) displaying plexiform fibrolamellar bone (centre) and dense Haversian bone (bottom). Note that the medullary cavity (bottom) has been subsequently closed by the deposition of endosteal lamellar bone which was in turn replaced by secondary Haversian bone. Anterior at the bottom. **c)** Dense Haversian bone in a metacarpal of adult *Megaloceros giganteus* specimen NMING: F22534/4 (xpl, scale bar 0.5 mm).

Perinatal specimens of *Candiacervus* show no signs of bone remodelling. In general, resorption of primary bone and deposition of secondary osteons in cervid long bones starts in juveniles (Figure 5a). Large areas of Haversian bone in adults indicate strong bone remodelling during ontogeny. Apart from the femora, which have a mainly circular outline in cross section, Haversian bone is most dense where the curvature of the cortex is greatest, but in all long bones and specimens the area most affected by remodelling is the posterior area of the cortex.

In the femora, remodelling starts in the juvenile *Candiacervus* specimens with scattered secondary osteons in the middle cortex, mainly in its posterior part. Adult femora of all deer species sampled show strong remodelling (i.e. Haversian bone) in the posterior part, obscuring the growth record in this area of the bone. Remodelling is strongest in the cortical area of the linea aspera.

Similar to the femora, remodelling in the humeri of juvenile specimens of *Candiacervus* and *Dama* starts in the middle zone of the medial part of the cortex (Figure 5a). Adult humeri of all deer groups sampled show more remodelling than the femora. Nevertheless, the amount of remodelling is low enough to leave a sufficient growth record.

Tibiae of juvenile *Candiacervus* and *Dama* start being remodelled mainly in the medial and lateral parts of the middle and inner cortex, leading to the deposition of dense Haversian bone (Figure 3d). In rare cases, dense Haversian bone is also found in the outermost part of the cortex in *Megaloceros*. Again, however, the amount of remodelling is low enough to leave a sufficient growth record.

Haversian bone in juvenile radii of *Candiacervus* and *Dama* indicates an early onset of secondary remodelling in the inner cortex. Strong remodelling in adult radii of *Candiacervus*, *Dama*, and *Megaloceros*, especially in the posterior area of the inner cortex, obscures the growth record to a large degree in these bones.

Ulnae of all juvenile deer species sampled are already remodelled to a high degree, especially in the inner cortex surrounding the medullary cavity, indicated by dense Haversian bone. Adult ulnae are strongly remodelled leaving only small areas of primary bone tissue in the posterior part of the cortex. During ontogeny, the medullary cavity shifts to the anterior area of the cortex being subsequently closed by the deposition of endosteal lamellar bone which is in turn subsequently replaced by dense Haversian bone (Figure 5b). Due to this strong remodelling of the ulnae in all deer species sampled, and since only small areas of primary plexiform bone tissue are left in the bone cortex, skeletochronological interpretations are not feasible.

Remodelling in metapodials begins with the development of Haversian bone in the inner cortex and is already strongly developed in juvenile specimens. In all specimens, the area most affected by remodelling is the posterior area of the cortex. Adult deer metapodials are strongly remodelled, occupying about half of the cortex and obliterating the growth record by development of dense Haversian bone (Figure 5c).

### Skeletochronology and growth mark analysis

Cyclical growth patterns have been observed in many extant artiodactyls [41]. However, in mammals bone resorption and remodelling may occur throughout ontogeny and LAG counts and age are apparently decoupled in old individuals [40,42,43]. Therefore, individual ages are often underestimated by bone histological studies, making cementum analysis a crucial tool in order to study longevity in fossil cervids (see also Additional file 1: Discussion).

LAGs are present in all deer taxa sampled. Femora, tibiae, and humeri of adult specimens show, due to relatively low remodelling, the highest LAG counts. The maximum LAG counts seen in adult femora are eight in dwarf *Candiacervus* (n = 3, Figure 2c), six in *Dama* (n = 2), and 10 in *Megaloceros* (n = 2).

In order to quantify growth rates in the sampled deer taxa, we measured non-remodelled cortical growth zones until the “virtual end of circumferential bone growth” [42]. Growth marks in the outermost part of the bone cortex, not giving a signal because of similarity and diminutiveness of growth zone thickness, have been omitted. It has recently been shown that in antelope (*Addax nasomaculatus*) femora the first LAG is resorbed during ontogeny [39]. Ruminants such as antelopes and cervids show similar long bone morphology as well as similar arrangement of bone tissue types, bone remodelling, and resorption patterns [41,58]. Superimposition of sections of femora and tibiae of perinatal, juvenile, and adult dwarf *Candiacervus,* juvenile and adult specimens of *Procervulus praelucidus*, and a juvenile as well as two adult specimens of known-age *Dama dama* however indicate that no LAG is lost during ontogeny in femora and tibiae of these cervids. On the grounds of phylogenetic parsimony we consider it as justified to assume that in general bone resorption patterns are identical throughout cervids. This approach made retrocalculation techniques as performed for dinosaurs dispensable [12,59]. In order to make growth rate measurements comparable, we numbered the growth zones of adult specimens starting with two since the first growth zone is at least partially resorbed and not available for skeletochronology (Figure 3b,c,d).

Additionally, and in order to verify our observations made by growth zone counts and measurements, we followed [51] in determining how fast dwarf *Candiacervus*, *Megaloceros,* and *Procervulus* grew over a hypothetical 365 days growth period by assessing growth rates *sensu* Sander & Tückmantel and comparing them to the values observed in extant cervids. Although bone growth rates per day are always approximations, they allow comparison to and verification of known bone apposition rates in extant and fossil vertebrates [51,60].

**Figure 6:**
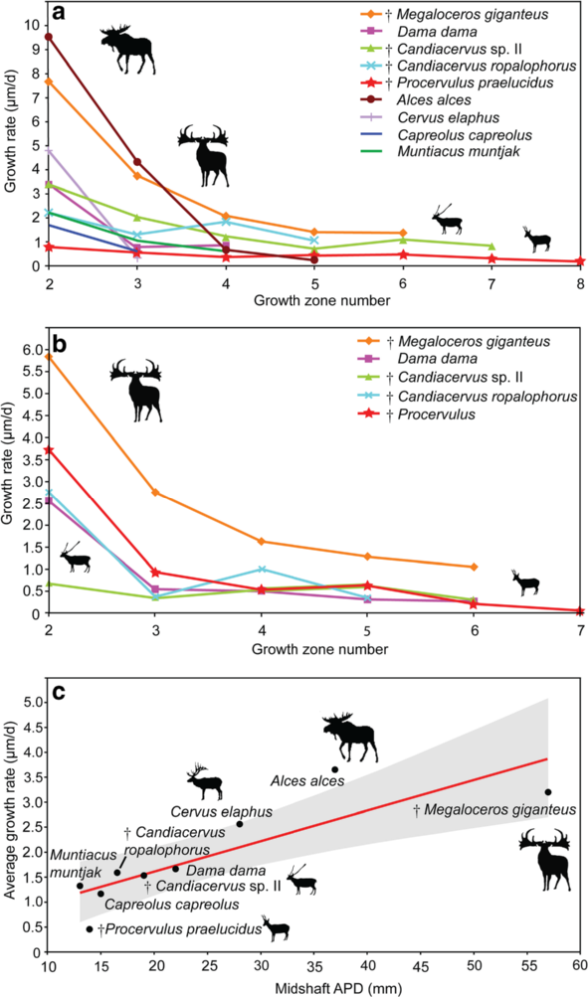
**Cervid growth rates. a)** Graph of growth zone measurements of cervid femora sampled. Points indicate sample means or measurements of single specimens (see also Additional file 2). Note exceptionally high growth rates in the first two growth zones of *Alces* and *Megaloceros* as well as exceptionally low rates of *Procervulus*. Growth zones numbered, starting with two for the innermost complete zone of the cortex. **b)** Graph of cervid tibiae sampled. Note the eight times higher growth rate in growth zone two of *Megaloceros* compared to *Candiacervus* sp. II (and still twice as high as in *C. ropalophorus*). **c)** Regression of average growth rates in cervid femora (*n* = 12, *r* = 0.85111, *p* = 0.0036142.). Shaded region represents the 95% confidence interval. Anteroposterior diameter (APD) of femoral midshaft region is taken as proxy for body mass.

In contrast to dwarfed forms of *Candiacervus* and to *Dama*, *Megaloceros* femora indicate up to five times higher growth rates, with the second growth zone yielding a rate of 7.69 μm/d, the third one 3.69 μm/d, and growth zones four to six between 2.04 – 1.35 μm/d (Figure 6a). The femora of *C. ropalophorus* indicated a growth rate of 2.19 μm/d to 1.81 μm/d in growth zones two to four (Figure 6a). In the following year the growth rate slightly decreased to 1.04 μm/d. The growth rate of *Candiacervus* sp. II decreased from 3.34 μm/d in growth zone two to 1.19 μm/d in growth zone four. In growth zones five to seven the growth rate ranged from 0.69 μm/d to 0.81 μm/d. The femoral growth rate of *Dama* is higher than that of *C. ropalophorus* and equal to that of *Candiacervus* sp. II in the second growth zone (3.34 μm/d). After that, growth rate strongly decreased below the rates of *C. ropalophorus* and sp. II (0.73 and 0.84 μm/d).

The growth rates recorded in the tibiae are similar to the ones obtained for the femora. *C. ropalophorus* grew in zone two at a rate of 2.47 μm/d, whereas *Candiacervus* sp. II only grew at 0.69 μm/d (Figure 6b). *D. dama*, at 2.54 μm/d, occupies an intermediate position between the dwarfed deer and *Megaloceros* (5.81 μm/d). Growth rate strongly decreases from zone two to zone three in most deer species sampled: 2.76 μm/d in *Procervulus*, 3.07 μm/d in *Megaloceros* but only 0.34 μm/d in *C.* sp. II. There is discrepancy among taxa, and *C. ropalophorus,* although the smallest species, shows about four times higher growth rate in the second growth zone (similar to *Dama*) compared to *Candiacervus* sp. II (Figure 6b). This demonstrates the diversity of life history parameters across morphotypes of *Candiacervus* in Crete during the Pleistocene [61,62].

Average growth rates of 0.46 μm/d (Figure 6c) in femora of *Procervulus* were the lowest measured for all the deer taxa sampled, lying below the lower limit of their 95% confidence interval. *Muntiacus*, *Capreolus*, *Dama* and dwarf *Candiacervus* show average to low growth rates around 1.4 μm/d, whereas *Cervus elaphus* (2.58 μm/d) had distinctly higher growth rates lying on the upper limit of the 95% confidence interval. *Alces alces* shows with 3.68 μm/d the highest average growth rates. In contrast, the absolute high growth rates (based on the growth zones preserved in the cortical bone tissue) of *Megaloceros* are relatively low given the regression (Figure 6c, average 3.23 μm/d), but still within the limits of the 95% confidence interval.

**Figure 7:**
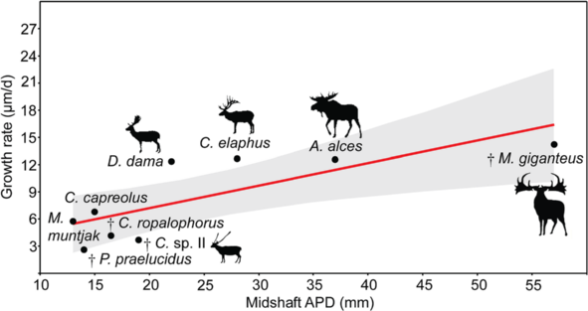
**Regression of growth rates** ***sensu*** **Sander & Tückmantel in cervid femora (** ***n*** **= 12,** ***r*** **= 0.78168,** ***p*** **= 0.012835).** Shaded region represents the 95% confidence interval. Anteroposterior diameter (APD) of femoral midshaft region is taken as proxy for body mass (see also Additional file 2).

Growth rates *sensu* Sander & Tückmantel plotted against the anteroposterior bone diameter as a proxy for body mass indicate three groups (Figure 7): A group with high growth rates including *Megaloceros* (14.22 μm/d), *Cervus elaphus* (ZIUK 23517; 12.66 μm/d), *Alces* (ZMUZ 20242; 12.58 μm/d), and *Dama* (12.35 μm/d); an intermediate group with *Capreolus* (ZIUK 9872; 6.79 μm/d) and *Muntiacus* (ZIUK 7994; 5.75 μm/d); and a group showing low growth rates, including *Candiacervus* sp. II (PIMUZ A/V 5218, 3.7 μm/d), and ranging from 4.16 μm/d in *Candiacervus ropalophorus* to 2.6 μm/d in *Procervulus* (Figure 7). *Dama* and *Cervus elaphus* plot above the upper limit of the 95% confidence interval whereas only *Candiacervus* sp. II lies well below the lower limit of the 95% confidence interval. All other cervids sampled show growth rates within the 95% range given their body size (see also Additional file 1: Discussion).

### Skeletal maturity estimates

Examination of femora of extant cervid taxa revealed the occurrence of OCLs not coeval with the timing of sexual maturity, as reported for femora of antelopes [39]. An adult specimen of *Dama dama* (ZIUK 9630; Figure 3b) shows three LAGs before the OCL, in contrast to the onset of sexual maturity which has been reported to occur during the second year of life in *Dama dama* [63] (Figure 1a). These observations suggest that the transition of the fibrolamellar complex (FLC) to the OCL, which is not clearly definable in every specimen, is indicating cervid skeletal maturity *sensu* [64] and not sexual maturity. This is well in accordance with known data of skeletal maturity for *Dama* [30,31], and a recent study on growth marks in the bone tissue of ruminants that examined cervid bone histology in detail [41]. The bone cortex of dwarf *Candiacervus* femora indicates skeletal maturity at five to seven years whereas *Megaloceros* reached skeletal maturity at five to six years. One *Procervulus* specimen indicates attainment of skeletal maturity at seven years whereas *Cervus elaphus* (four to six years) ranges with its timing of skeletal maturity between *Megaloceros* and *Alces* (three years) [31].

### Cementum analysis and longevity

**Figure 8:**
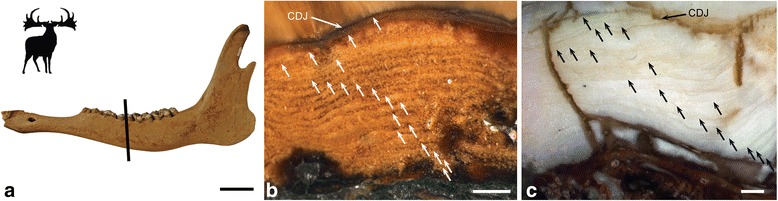
**Cervid tooth histology. a)** Senescent *Megaloceros* specimen PIMUZ A/V 2235. Left mandible in lateral view. Black bar indicates plane of sectioning. Scale bar, 50 mm. **b)** Tooth cementum of same specimen showing 19 rest lines (white arrows). Scale bar, 0.5 mm. **c)** Tooth cementum of the upper first molar of *Candiacervus* sp. II (PIMUZ A/V 5241) showing 18 rest lines (black arrows). CDJ = Cementum-dentine junction. Scale bar, 0.5 mm. Direction of cementum apposition to the bottom.

Tooth cementum analysis of first molars of *Candiacervus* provided an age of four years for a juvenile *Candiacervus* sp. II and an age of at least nine years for an adult specimen of *C. ropalophorus*. Two senile *Megaloceros giganteus* specimens revealed ages of 16 and 19 years (Figure 8a,b). Rest lines in two old *Candiacervus* specimens gave ages of 12 (dwarf *Candiacervus* sp.) and 18 years (*Candiacervus* sp. II, Figure 8c). Dwarf *Candiacervus* thus, in an allometric context, show an extended lifespan compared to other deer of similar body size such as *Mazama* with a maximum longevity of 12 years in the wild (Figure 1a). This is well in accordance with observations of a recent study on population structure and dynamics in dwarf *Candiacervus* [62].

A positive linear relationship between body mass and longevity has been demonstrated in bats and mammals in general [47,65], although this is difficult to assess in our cervid data set (Figure 1b) because of issues of comparability: for example, the 32-year maximum age of a captive specimen of *Cervus elaphus* [63], a farmed and extensively studied species, is probably anomalously high. Conversely, *Megaloceros* appears short-lived when body mass is taken into account (Figure 1b), although the sample size was small. Clearly, there is much diversity in life history across deer species, and examination of other populations of *Megaloceros* may reveal more diversity in the giant deer than we have recorded in our study [18,19].

## Conclusions

Our histological observations indicate lower growth rates in dwarf *Candiacervus* than in *Megaloceros*. The presence of laminar bone tissue in the middle and outer cortex of adults of small-sized deer (dwarf *Candiacervus, Procervulus* and *Muntiacus*) suggests lower growth rates, in contrast to the occurrence of plexiform bone in intermediate to large sized forms. Growth rates determined by growth zone measurements in femora and tibiae indicate comparable growth rates of intermediate sized and small deer species, with slower growth in the stem group cervid *Procervulus*. Growth rates in the two small *Candiacervus* morphotypes are different, underscoring the flexibility of growth strategies and the importance of a resolved phylogenetic framework to study heterochrony. Skeletal maturity data suggest late maturation for dwarf *Candiacerus* and *Procervulus* in comparison to a similarly small cervid such as *Muntiacus* attaining skeletal maturity in two years [32].

The landmasses of islands have been hypothesized of being able to support only a limited number of primary producers affecting the energy flow at higher trophic levels. As a consequence, energy-poor islands are expected to be impoverished in competitors and predators making especially high growth rates and high reproductive rates dispensable to unnecessary [32,46,66-68]. A delay in the attainment of maturity was recorded for the dwarfed island bovid *Myotragus balearicus* [46], and was thought to be associated with synchronisation of metabolic requirements to fluctuating resource levels. The delay of attainment of maturity in the island cervid *Candiacervus* and the continental *Procervulus* demonstrates the variability of life history parameters in island as well as continental cervids. This might point towards fluctuating resource levels in the Late Pleistocene Crete, selecting for a growth pattern recalling that of the stem-cervid *Procervulus*.

The oldest individual seen in our cementum analysis of *Megaloceros* was 19 years, comparable to maximum longevity in extant *Dama.* This find extends an age based on cementum analysis [25] by five years and lies below another estimate [69] also based on cementum analysis, by four years. However, [69] did not illustrate cementum rest lines of the specimen studied. We therefore consider the result of our cementum analysis as the highest rest line count in *Megaloceros*. The oldest individual seen in our cementum analysis of *Candiacervus* was 18 years, indicating prolonged longevity for a deer of this body size.

The exact persistence time of the *Candiacervus* radiation on Crete is not known but was apparently much shorter, i.e., less than 0.5 myrs [5], compared to *Myotragus balearicus*, which dwelt on Majorca for 5.2 myrs [46]. The less extreme modification of bone tissue observed in dwarf *Candiacervus* could be related to shorter persistence time and perhaps to the larger size of Crete [3,8].

In life history theory, slow-developing long-lived species are typically associated with low fecundity and rapidly-developing short-lived species with high fecundity [36,70]. The condition found in *Candiacervus* has features in common with that of *Myotragus* [46], but is achieved with less far reaching modification of bone tissue, as indicated by the absence of lamellar-zonal bone throughout the cortex. Neither does the *Myotragus* pattern occur in the pygmy mammoth, *Mammuthus exilis*, from Santa Rosa Island, California, whose bone cortex is characterized by laminar fibrolamellar bone [71]. Therefore we suggest variable modes of life history and size evolution among island mammals in line with [65-68,72].

### Availability of supporting data

The data sets supporting the results of this article are included within the article (and its additional files).

High resolution versions of the histological figures provided in this article are available on MorphoBank [73], Project P2083 (http://www.morphobank.org**/**permalink/?P2083).

## Additional files

Additional file 1:
**Includes Table S1, additional discussion on individual age estimates and growth rates, additional information on methods used in this study, additional references, and Figure S1.**
Additional file 2:
**Primary data for growth rate analysis: Growth zone measurements, mean species growth rates, average species growth rates, bone diameter, OCL thickness, and number of non-OCL lines of arrested growth (for growth rates** ***sensu*** **Sander & Tückmantel).**
