# Supplementary material for: Growth in fossil and extant deer and implications for body size and life history evolution
Source: BMC Evol Biol. 2015 Feb 14;15:19. doi: 10.1186/s12862-015-0295-3 (PMC4332446; doi:10.1186/s12862-015-0295-3)
Supplement: Additional file 1: — Includes Table S1, additional discussion on individual age estimates and growth rates, additional information on methods used in this study, additional references, and Figure S1. [file 12862_2015_295_MOESM1_ESM.pdf]

Additional file 1

Table S1: Histological traits of cervid taxa sampled. The terminology follows [57].

Histological traits	Small body size	Intermediate/large body size
Inner cortex (excl. inner cortical layer)		
Primary bone	fibrolamellar	fibrolamellar
Vascularization	plexiform	plexiform
Lines of arrested growth	none	none
Middle cortex		
Primary bone	fibrolamellar	fibrolamellar
Vascularization	plexiform-laminar	plexiform/radiating
Lines of arrested growth	present	present
Outer cortex (excl. outer circumf. layer)		
Primary bone	fibrolamellar	fibrolamellar
Vascularization	laminar/avascular	plexiform/radiating/longitudinal
Lines of arrested growth	present	present
Remodelling	strong	strong
Resorption/Endosteal bone	present	present
Haversian bone	normal to dense	normal to dense

Additional discussion

Individual age estimates

In order to explore the issue of underestimation of individual ages by bone histological studies, we performed age determination based on both tooth cementum and bone histological analysis for two specimens of *Dama*. The growth record of an eight year old specimen (ZIUK 9630), of which the age was determined by cementum analysis, is represented by six LAGs in the bone tissue including LAGs in the OCL. Another eight year old specimen (PIMUZ A/V 5248) shows only two LAGs in total. This confirms that age estimates from growth marks in bone tissue variably underestimate the actual age.

Growth rates

Growth rates *sensu* Sander & Tückmantel give in some regards a different picture than average growth rates do (Figure 6c, Figure 7). *Alces* (ZMUZ 20242) lies well within the 95 % interval which is not the case in average growth rates. The fact that *Megaloceros* shows a slightly larger value compared to *Alces*, *Cervus elaphus* (ZIUK 23517) and *Dama* indicate the hypothetical nature of growth rates *sensu* Sander & Tückmantel and suggest the reflection of differences in number of LAGs and bone diameter but not actual patterns of growth. The same probably applies to the fact that *Muntiacus* (ZIUK 7994) shows much higher values in maximum growth rates than dwarf *Candiacervus*.

Additional methods

Age classes in long bones have been determined by the state of epiphyseal fusion [31] and proximodistal length. For selection of aged specimens to be sampled for cementum analysis, we used known tooth eruption/wear state patterns in extant cervids [31, 74-76, Kolb & Azorit unpublished observations]. The bone tissue of perinatal, juvenile, and adult dwarf *Candiacervus* (*C. ropalophorus* and *Candiacervus* sp. II) are described. The observations are compared to the bone tissue of adult *Megaloceros giganteus* (extinct), juvenile as well as adult *Dama dama*, adult *Procervulus praelucidus* (extinct), *Muntiacus muntjak*, *Capreolus capreolus*, *Cervus elaphus*, and *Alces alces* specimens. The histological terminology used follows [57].

Due to differences in growth rates *C. ropalophorus* and *Candiacervus* sp. II were treated separately unlike our approach to bone tissue analysis. Points of growth zone measurements are based on one specimen each for *Candiacervus* sp. II, *Procervulus*, *Muntiacus*, *Capreolus*, and *Cervus* in the femora (Figure 6a, c). In the tibiae, points represent values of the mean of three specimens for *Megaloceros*, whereas *Candiacervus* sp. II, *Muntiacus*, *Capreolus*, and

Cervus are represented by one specimen each (Figure 6b). All other points represent mean values of two specimens.

Average growth rates represent average values of measured growth zones. Attained values of specimens have then been averaged in order to obtain an average value for each species. For attaining growth rates *sensu* Sander & Tückmantel [51] the radius of the bone was measured along the anterior quadrant of the bone (excluding the OCL) and divided by the number of LAGs excluding those in the OCL. This method assumes that the entire thickness of the bone from the center to the first growth line was formed within the first year of life. Prenatal bone apposition is not taken into account and, following [51], the OCL and contained LAGs are excluded. Since the original term "maximum growth rates" [51] might be misleading in the context of growth period estimates and their application for growth rate calculations, we use the "term growth rates *sensu* Sander & Tückmantel". After attaining growth rates *sensu* Sander & Tückmantel expressed as µm/d and assuming a yearlong growth period for each specimen, values have been averaged for attaining mean growth rates *sensu* Sander & Tückmantel of each species studied.

Additional references

74. Chapman D, Chapman N: *Fallow deer - their history, distribution, and biology*. Machynlleth: Coch-y-bonddu books; 1997.
75. Azorit C, Analla M, Carrasco R, Calvo JA: **Teeth eruption pattern in red deer (Cervus elaphus hispanicus) in southern Spain**. *Anales de Biologia* 2002, **24**:107-114.
76. Azorit C, Analla M, Carrasco R, Munoz-Cobo J: **Determinación de edad por desgaste dental en el ciervo ibérico (Cervus elaphus hispanicus)**. *Bol R Soc Esp Hist Nat (Sec Biol)* 2003, **98**(1-4):123-134.

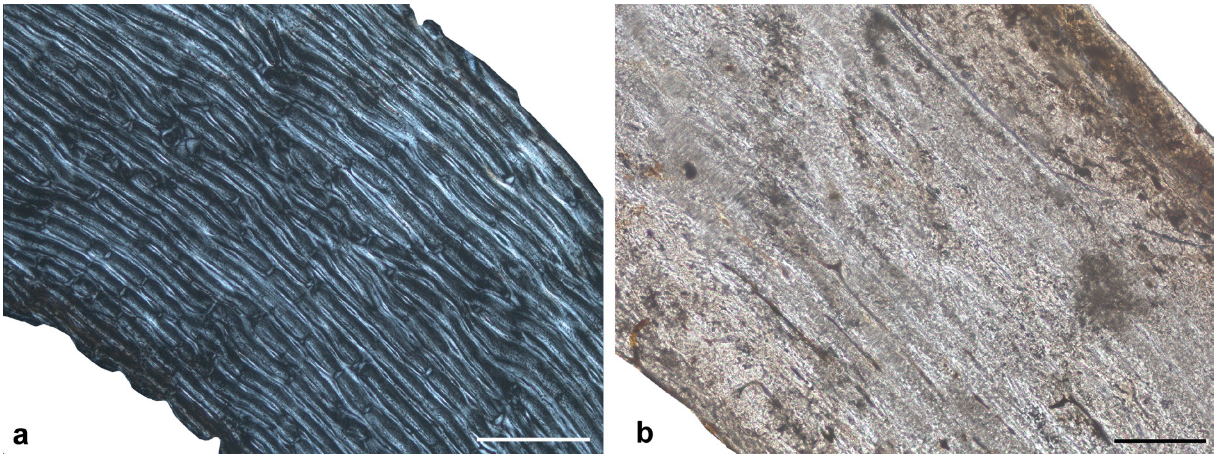

Figure S1. Femoral bone cortex of *Dama* and *Procervulus*. Typical plexiform bone tissue in a) juvenile *Dama* specimen PIMUZ A/V 5249 (xpl, scale bar 0.5 mm) and low amount of vascularisation in b) adult *Procervulus* specimen BSPG 1937 II 23226 (lpl, scale bar 0.2 mm). Brown and dark grey areas in the *Procervulus* specimen represent areas of recrystallisation. Bone surfaces at top right.
